# Neurofilament light: a narrative review on biomarker utility

**DOI:** 10.12703/r/10-46

**Published:** 2021-05-07

**Authors:** Shilpa Narayanan, Akshay Shanker, Tanvi Khera, Balachundhar Subramaniam

**Affiliations:** 1 Department of Anesthesia, Critical Care, and Pain Medicine, Beth Israel Deaconess Medical Center, Harvard Medical School, Boston, MA 02215, USA; 2 The Lewis Katz School of Medicine at Temple University, 3500 North Broad Street, Philadelphia, PA 19140, USA

**Keywords:** Neurofilament Light, NfL, biomarker, neuroinflammation, neurodegeneration, delirium, cardiac surgery

## Abstract

Neurofilament light (NfL) is a scaffolding protein that is located primarily within myelinated axons and that provides increased conduction speed and structural support. In recent years, NfL has been used as a disease biomarker on the basis of the observation that axonal injury results in elevated levels of NfL in cerebrospinal fluid or blood. This review focuses on how cerebrospinal fluid and plasma NfL have been studied in various disorders such as Alzheimer’s disease (AD) and multiple sclerosis (MS) in relation to neuroinflammation and cognitive dysfunction. Focusing on the role of NfL as a biomarker for AD and MS, this review aims to further explore the potential of NfL as a promising biomarker with regard to surgery- and anesthesia-based incidents for postoperative cognitive decline and delirium. A search of the PubMed database yielded 36 articles, 31 of which are from within the last 3 years, that show how NfL has been observed and studied under various types of trials and disease cohorts and potential future directions. Higher levels of NfL have frequently been correlated with disease progression and prognosis of AD and MS, and delirium has been found to share a neuroinflammatory pathophysiology that NfL could help to measure. Focusing on NfL as a biomarker for neurodegenerative decline, these studies indicate that the protein could be further tested and related to postoperative aspects that result in cognitive dysfunction, and it has the potential to be an established delirium biomarker, particularly in the realm of the perioperative course.

## Introduction

Neurofilaments (NFs) are cylindrical and elastic proteins located in neurons that provide structural stability and dictate their asymmetrical shape. NFs are found in dendrites and the neuronal soma though not as abundantly as in myelinated axons^1,2^. NFs promote the radial growth of axons and are expressed more in larger myelinated axons, promoting a higher conduction velocity^1–3^. NFs are released upon axonal injury, leading to an increase in concentration in cerebrospinal fluid (CSF) and blood. Among the four NF subunits, neurofilament light (NfL) is the most abundant and soluble in CSF and blood. In particular, NfL has served as an important biomarker for many neurodegenerative diseases such as multiple sclerosis (MS), Alzheimer’s disease (AD), and Parkinson’s disease (PD)^4–7^. NFs have been measured through CSF for both central nervous system and peripheral nervous system diseases, although this requires a lumbar puncture, which is a more invasive procedure. Similar to the other subunits of NFs, NfL can be measured in CSF through the antibody–antigen interactions seen in enzyme-linked immunosorbent assay (ELISA) technology^2,4^. However, this method is neither sensitive nor specific enough for quantifying levels of NfL in blood since the concentration of NfL in blood is far lower than in CSF. More recent advances in immunoassay technology have contributed to the ability to measure NfL in blood. In particular, the quantification of NfL concentration and detection of longitudinal changes in NfL levels specific to plasma have been achieved via single-molecule array (Simoa) technology^2,4–6^. Many studies have shown a positive correlation between the plasma or serum and the CSF concentration of NfL, demonstrating the effectiveness of NfL as a blood biomarker for multiple diseases^2,5,6^.

For this narrative review, we specifically looked at the function of NfL and aimed to address its use in previous studies and trials, particularly with regard to neurodegeneration, neuroinflammation, and neuronal injury as a result of surgery and anesthesia. Studies pertaining to cognitive decline and delirium were also incorporated to emphasize the relationship between NfL and perioperative neurocognitive disorders (PNDs). However, it should be noted that there is an ongoing discussion regarding the definition of PND as a more overarching term for events or diagnoses of cognitive dysfunction and decline as a result of surgery and anesthesia^7^. A literature search was conducted on PubMed with defined keywords, including “neurofilament light”, “NfL”, “neurodegeneration”, “neuroinflammation”, “cognitive dysfunction”, “postoperative delirium”, and “perioperative neurocognitive disorders”. Scientific papers and reviews included were published between 2011 and 2020, and over 25 of the references were published in the last three years.

## Neurofilament light and neurodegenerative disorders

The measured levels of NfL have allowed the assessment of disease onset, progression, and prognosis in neurodegenerative diseases. NfL is released at a constant and low level from axons under normal conditions; however, this rate increases with age. During events of axonal damage, concentrations of NfL in CSF can increase to 40 times their original level^4,6^. The observance of this increase in NfL levels therefore can serve as an indication of the degree of axonal damage and disease severity. In MS, for example, axonal injury occurs during the early phases and degeneration continues as the disease progresses, providing NfL with prognostic potential. This degeneration correlates with NFL levels in affected patients, and greater levels are measured in the relapsing–remitting disease course^4,8^. Additionally, NfL has been found to be sensitive to treatment for MS, and a higher percentage decrease is seen in patients who receive treatment as compared with healthy control groups^9^.

In regard to neurodegeneration, both neurocognitive decline and neuronal loss associated with AD correlate with increased levels of plasma and CSF NfL; a peak in the rate of increase is on par with the onset of AD^4–6,10,11^. A 2019 longitudinal study found that the rate of change in serum NfL levels served as a predictor for presymptomatic AD but that absolute NfL levels were observed to indicate the symptomatic phase and progression of AD^10^. Although the study demonstrated that a greater rate of change would indicate a higher conversion from presymptomatic to symptomatic AD, the optimal disease period at which the rate of change is most accurate, a characteristic that would further the ability of NfL as a biomarker, was not determined^10^. Similarly, a correlation between higher plasma NfL levels and poor cognition in PD has been studied^11^. However, no relationship between the motor symptoms and the biomarker was established, emphasizing the specificity of NfL to cognitive dysfunction in diseases^11^.

## Neurofilament light in perioperative neurocognitive disorders

### Acute neurofilament light changes following surgery

**Figure 1.**
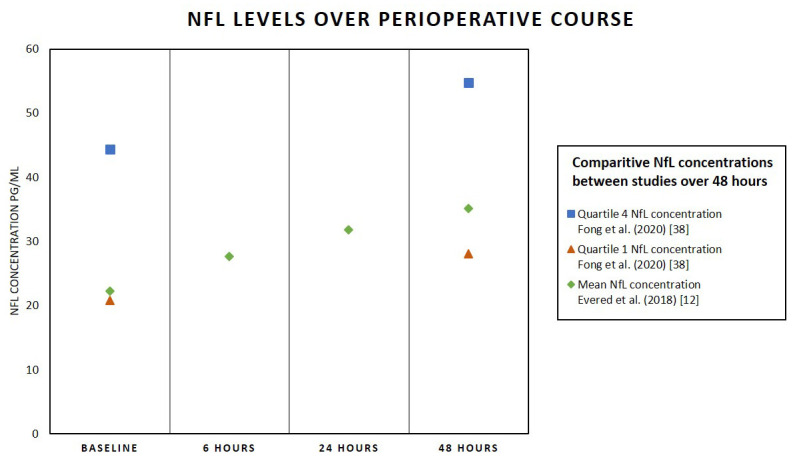
Neurofilament light (NfL) levels over perioperative course. The figure shows a comparative sequential increase in NfL in patients 60 years or older, for a period of 48 hours postoperatively, as described by Evered *et al.* and Fong *et al.*, respectively^12,15^. Mean plasma neurofilament light concentration measured at baseline, 6 hours, 24 hours, and 48 hours for patients undergoing hip or knee surgery under general anesthesia (Evered *et al.*^12^). Baseline and 48-hour NfL concentrations measured in patients undergoing major elective surgery (Fong *et al.*^15^). Quartile 1 is the lowest median quartile range, defined by NfL concentration of no more than 20.76 pg/mL at baseline and perioperative neurocognitive disorder 28.08 pg/mL at postoperative day 2. Quartile 4 is the highest median, defined by NfL concentration of at least 44.39 pg/mL at baseline and at least 54.76 pg/mL at postoperative day 2.

Changes in NfL levels have also been observed in response to surgery and anesthesia, suggesting an association between neuronal damage and surgery as measured by the biomarker through the perioperative course^12–14^. The aims of a 2018 study by Evered *et al*. were to observe and investigate the effects of anesthesia and surgery on cognition through the use of the neuronal injury biomarkers tau and NfL^12^. The cohort consisted of 30 patients who were at least 60 years old and who were undergoing either hip or knee arthroplasty with general anesthesia (combined with either bupivacaine or ropivacaine). Blood samples were collected over the perioperative course at five different time points: before surgery and 30 minutes (for 17 participants), 6 hours, 24 hours, and 48 hours after the surgical incision. The analysis compared change over time from the baseline of each individual and showed a significant and sequential increase in plasma NfL concentration at up to 48 hours (Figure 1). These results demonstrated a more acute response to neuronal injury rather than chronic neurodegeneration due to surgery and anesthesia^12^.

### Postoperative delirium

Delirium is defined by the *Diagnostic and Statistical Manual of Mental Disorders, Fifth Edition* (DSM-5) as an acute onset or fluctuating course of inattention along with either disorganized thinking or an altered level of consciousness. Delirium has a high incidence in the postoperative period, especially in elderly patients undergoing cardiac surgery^13,14,16^,^17^. In the perioperative period, there are various risk factors for delirium, including preoperative cognitive status, elderly age, untreated acute pain, excessive use of sedatives, and opioids in the postoperative period. Although the underlying pathophysiology of delirium is not fully understood, there are two leading hypotheses: dysregulation of inflammation and impairment in neurotransmission^13,16,17^. Various biomarkers have shown an association of systemic inflammation with delirium severity and duration, but current literature falls short in providing a reliable biomarker for predicting the incidence of delirium^18–21^. However, studies have linked CSF biomarkers to delirium, and the neuroinflammation pathophysiology found in AD is associated with delirium^22^. Thus, it may be of value to study the trends of NfL as a screening modality for imminent onset of postoperative delirium.

### Delirium, neurofilament light, and other biomarkers

Because delirium results in cognitive changes, investigators have explored links to NfL, specifically those having to do with cognition and neuronal damage. Previous candidate biomarkers measured for delirium have included tau and interleukin-8 (IL-8)^12,18,23,24^. Postoperative fluctuation in tau levels (a rapid increase followed by a decrease in the first 48 hours) points to the neuronal response to surgery and anesthesia; however, this fluctuation lacks a longitudinal correlation with the incidence of delirium^12,23,24^. Even though the cytokine IL-8 has demonstrated a correlation with severity of delirium through inflammation, it is difficult to determine whether surgery independently impacts inflammation or delirium and, if so, to what extent^24^. NfL has been directly linked with cognitive impairment and neurodegeneration, and the former has also been associated with postoperative delirium^4,16,25^. Casey *et al*. established NfL as a biomarker for neurodegeneration by correlating increased levels of plasma NfL with patients positive for neurodegeneration on neuroimaging^18^. The same study also demonstrated a rising trend of NfL in a postsurgical population with a peak on postoperative day 1 in delirium-positive patients when compared with non-delirious controls. This positive correlation was seen even if the patients were found to be delirious on a later postoperative day, suggesting that the biomarker could be predictive for the state of postoperative delirium^18^. The link between neuronal damage and delirium was strengthened in that study by showing independence from the pathophysiologic role that inflammation has been hypothesized to play in delirium^17–19^.

The cardiac surgery population is associated with a high (up to 50%) incidence of postoperative delirium along with worse postoperative outcomes^18,20,26,27^. A recently published case series tested the association of NfL levels on three groups of cardiac surgery patients grouped by procedure (off-pump cardiac arterial bypass or procedure with cardiopulmonary bypass) and delirium status: without developing delirium in off-pump patients, without delirium, and postoperative delirium in cardiopulmonary bypass patients^13^. All groups had an increased level of NfL in the postoperative period when compared with the baseline values, and the concentration was strikingly higher in the delirious patients who underwent cardiopulmonary bypass intraoperatively compared with the no-delirium and evolving-delirium groups^24^. With a total length of hospital stay between 10 and 30 days for this group, the peak NfL levels were observed around the time of discharge, indicating the longitudinal value of the biomarker and the role it has as a diagnostic and monitoring tool in the perioperative course^24^. However, the case series format of that study limits study validity as there was no randomization and it is unclear whether NfL was an associated or causal factor in the delirium pathway. Therefore, there is a need for matched cohort studies or randomized control trials to further analyze the relationship between NfL and postoperative delirium in cardiac surgery. Alifier *et al*. investigated the relationship between neuronal injury and surgery by measuring NfL concentrations in 25 cardiac surgery patients and 26 otolaryngeal surgery patients^28^. Samples were taken before, during, and after surgery, and NfL was found to increase in concentration over a period of 7 days after on-pump cardiac surgery compared with no changes in otolaryngeal surgery. This association between cardiac surgery and neuronal injury in patients indicates a key interaction in observing cognitive decline and PND, particularly through NfL^24,26–29^. Through the use of NfL as a biomarker, there is more potential for early detection of delirium in the postoperative period, possibly before the symptoms present themselves. Having a validated biomarker that can predict delirium would help clinicians take mandated precautionary measures and reduce the in-hospital morbidity of these patients, potentially using this biomarker to track the effectiveness of their measures over a period of time.

### Delayed cognitive recovery and mild cognitive impairment following surgery and neurofilament light

Cognitive dysfunction and decline have been frequently associated with surgery, particularly in relation to postoperative complications in cardiac surgery^26,27,29^. Conditions such as PNDs have been shown to contribute to decreased quality of life and increased risks of developing dementia and AD. In a 2018 randomized trial, Danielson *et al*. observed that a high dose of methylprednisone in cardiac surgery patients attenuated systemic inflammatory markers such as IL-6 but did not impact neuroinflammatory markers like NfL and tau^30^. It should be noted, however, that the CSF biomarkers were obtained only once after surgery because of the invasive nature of the lumbar puncture, and NfL was measured through CSF and methods such as ELISA may have not been sensitive enough to note the changes in levels when compared with plasma or serum^30,31^.

## Neurofilament light with other biomarkers

When comparing NfL with other biomarkers used for neurodegenerative disease, many studies have used NfL alone and in conjunction with more disease-specific biomarkers not only to further understand the progression of disease but also to evaluate the potential of NfL as a biomarker^32^. Dhiman *et al*. compared NfL with amyloid-beta (Aβ) and tau proteins in order to distinguish AD from mild cognitive impairment (MCI)^33^. The study used the CSF form for all three biomarkers and found that NfL was highly specific and sensitive in separating patients with AD from healthy controls. This capability was on par with that of tau and Aβ. However, the study also observed that higher accuracy in the diagnosis between AD patients and MCI patients was seen when combining the latter biomarkers with NfL^33^. Similarly, higher levels of biomarkers such as GFAP and YKL-40 are correlated with increased MS disease progression while NfL determined changes in brain volume and neuronal injury^34^. NfL was also found to have accurate diagnostic value in neurodegenerative dementias when used in conjunction with tau^35^. These results could be attributed to the fact that NfL is not a disease-specific biomarker but rather one that applies particularly to neurodegeneration and axonal injury conditions, providing a range of potential uses in various diseases^32–35^.

## Neurofilament light and neurocognitive assessments

NfL has also been evaluated as a biomarker through its comparison with neurocognitive assessments to determine the status of a given patient’s cognitive function. A 2019 prospective observational study paired NfL levels with a neuropsychiatric exam and the Confusion Assessment Method for the intensive care unit (CAM-ICU) to measure functional outcome of sepsis-associated encephalopathy in patients with septic shock^36^. A significant increase in NfL was found over a period of 7 days in patients with sepsis, compared with the healthy controls, and was found in both plasma and CSF. The mean NfL increase in CAM-ICU–positive patients was significantly larger than that of CAM-ICU–negative patients. Overall, poor functional cognitive outcome correlated with higher levels of NfL and with a significant association with shorter survival^36^. A cohort of hip fracture patients who underwent elective surgery was also assessed for delirium with the CAM assessment, and higher levels of both serum and CSF NfL were found in patients positive for delirium^37^. The study by Halaas *et al*. compared NfL between hip fracture patients and cognitively normal patients undergoing elective surgery and used the CAM assessment to help diagnose delirium^37^. Samples consisted of CSF and preoperative and postoperative serum and found elevated levels of NfL serum in hip fracture patients with delirium, which positively correlated with NfL levels in CSF. This study, however, did not measure NfL at various time points to reflect gradual change in NfL concentration in patients with cognitive decline, as seen in other surgery-related studies^37^.

Olsson *et al*. established similar results when comparing NfL with the Mini–Mental State Examination, Montreal Cognitive Assessment, and Dementia Rating Scale for cohorts of various neurodegenerative disorders^38^. Higher NfL levels at baseline directly correlated with poorer scoring on the tests as well as faster decline of cognition in subsequent tests^38^. The use of NfL in conjunction with these assessments highlights brain injury in patients, and the combination further represents the prognostic ability of the biomarker in neurological outcome^36–38^.

In 2020, Fong *et al*. used neurocognitive assessments and measured four biomarkers associated with neuroaxonal injury—NfL, GFAP, tau, and ubiquitin carboxyl-terminal hydrolase L1 (UCHL-1)—in plasma samples in order to determine their relationship with the incidence, severity, and duration of delirium^15^. The cohort was identified as two groups of patients undergoing major elective surgery: delirium cases (patients with peak delirium on postoperative day 2) and no-delirium cases (no delirium throughout the length of their hospital stay); 54 participants, all over the age of 70, were in each group. This cohort design (a case-control design) paired the two groups on the basis of multiple variables. This study combined the use of neurocognitive assessments over the perioperative course with the blood collection at three time points: preoperatively (baseline), postoperative day 2, and 1 month after hospitalization^15^. The analysis with the assessment and NfL found that patients with the higher levels of NfL at baseline had a significantly greater risk of developing delirium postoperatively. Similarly, participants who had higher NfL levels either preoperatively or on postoperative day 2 experienced a more severe delirium, and these increases in concentration were not found in the other biomarkers that were tested. This result was corroborated with higher CAM severity scores, and there was a correlation between peak delirium severity and the change in baseline and postoperative day 2 NfL levels^15^. The analysis divided patients into four median quartiles; the highest quartile (Q4) had an increased risk of delirium compared with the lowest quartile (Q1) (Figure 1). Finally, although it was noted that absolute NfL increased in all participants, the delirium group was found to have higher NfL levels that continued to increase at 1 month after hospitalization, compared with the baseline level. This result was also examined with general cognitive performance (composited through various other neurocognitive tests), and an overall cognitive decline was found in patients with elevated NfL levels after 1 month^15^. The combined design of using both neurocognitive assessments and measuring NfL levels demonstrates a more effective method in following the trajectory of delirium in patients. This can be further broadened and applied to other surgical cohorts and randomized trials in order to establish NfL as a predictive biomarker for delirium.

The previously mentioned study by Evered *et al*. included follow-up cognitive testing, but no data were incorporated into the analysis^12^. The combined use of cognitive testing and NfL measurements in surgery-based studies would also validate the effectiveness in determining PND and other cognitive disorders. Previous case studies following surgical patients through their length of stay to depict the change in NfL levels have been conducted^24^. Combining the measurement of NfL at various timepoints along with the administration of neurocognitive assessments during the perioperative course would allow for parallel observations as well as monitoring a potential aligned trajectory in cognitive change.^12,36,37^. The study by Fong *et al*. was the first to measure NfL levels in combined efforts with neurocognitive assessments at a follow-up period of 1 month^15^. The observation that elevated NfL levels at 1 month may attribute to ongoing cognitive decline post-operatively. This could be further tested and expanded upon in larger studies of different surgical cohorts, including cardiac surgery patients, as seen with the case series carried out by Saller *et al*.^24^. Further confirmation from future studies would illustrate that the trigger of delirium may lead to neuroaxonal injury with the potential of long-lasting cognitive dysfunction^15^. An important note to be made is that developing a similar study design for the measurement of NfL could then progress into randomized control trials, comparing normal patients in a surgical cohort to those undergoing an intervention, to diagnose and track the progression of cognitive disorders or postoperative delirium.

## Conclusions

NfL, a protein specific to axonal injury, has recently been found to be a rising biomarker for multiple neurodegenerative disorders. Studies have both incorporated and focused on NfL for diagnostic and prognostic purposes and expanded the function of the biomarker beyond neurodegenerative diseases. NfL also shows potential in regard to surgery and postoperative cognitive dysfunction and delirium. Higher levels of NfL seem to correlate with increased neurodegeneration and poorer cognitive outcomes. Although most studies have used NfL in conjunction with other disease-specific biomarkers, more studies using both randomization of groups and the inclusion of multiple time points closer to the time of injury should be conducted. Thereby, NfL would be established either as part of a causative pathway in these disorders or as a byproduct of neurodegenerative and neuroinflammatory processes. Additionally, the use of multiple time points for NfL samples in conjunction with neurocognitive assessments would help to further indicate the prediction, progression, and trajectory of postoperative delirium and cognitive dysfunction over the perioperative course. The sensitivity that NfL provides in these studies illustrates a promising future as an established and definitive biomarker specifically for PNDs.
